# Continuous Size-Based Particle Separation Using Inertial Force and Deterministic Lateral Displacement

**DOI:** 10.3390/mi17020194

**Published:** 2026-01-31

**Authors:** Yile Xie, Zichen Wang, Wenjia Xie, Jeong Min Oh, Chun Lai, Jingqian Zhang, Raymond H. W. Lam

**Affiliations:** 1 Department of Biomedical Engineering, College of Biomedicine, City University of Hong Kong, Hong Kong 999077, China; yilexie2-c@my.cityu.edu.hk (Y.X.); zcwang22-c@my.cityu.edu.hk (Z.W.); wenjiaxie2-c@my.cityu.edu.hk (W.X.); jeongmoh2-c@my.cityu.edu.hk (J.M.O.); clai222-c@my.cityu.edu.hk (C.L.); jzhang3325-c@my.cityu.edu.hk (J.Z.); 2 Shenzhen Research Institute, City University of Hong Kong, Shenzhen 518057, China

**Keywords:** particle separation, inertial force, deterministic lateral displacement

## Abstract

Continuous, label-free particle separation is essential for a broad range of biochemical and biomedical applications. Here, we present a microfluidic device that integrates inertial focusing and deterministic lateral displacement (DLD) within a compact channel architecture to achieve size-based particle sorting under laminar flow conditions. The design combines upstream curved channels for initial lateral positioning with downstream micropillar-embedded curved channels to enhance separation resolution. Theoretical analysis and numerical simulations were performed to optimize channel geometry and micropillar arrangement, predicting size-dependent lateral displacement driven by centrifugal forces and pillar-induced constraints. Experimental validation using glass beads of two distinct sizes (8 μm and 15 μm) demonstrated a separation efficiency exceeding 93% across a range of flow rates and particle concentrations. The device offers a simple, cost-effective, and scalable solution for passive particle sorting without external fields or labeling. The flexibility of the design configuration can be adapted for diverse applications, including extracellular vesicles, barcoded hydrogel particles, and engineered drug-delivery carriers.

## 1. Introduction

Recent advances in bead-based biochemical assays have further increased the demand for reliable microscale particle preparation methods. Many biological and clinical workflows rely on monodisperse magnetic beads [1], fluorescent microspheres, and/or antibody-functionalized beads [2] as carriers for biomolecule capture, immunoassays, nucleic-acid purification, and multiplexed sensing platforms. Preparing these bead populations with well-defined size distributions is essential for ensuring consistent binding capacity, reaction kinetics, and optical or magnetic signal output.

The rapid advancement of microfluidic technologies has transformed the landscape of biomedical research, diagnostics, and therapeutic applications [3]. Microfluidic systems enable precise manipulation of small liquid volumes, offering significant advantages such as reduced reagent consumption, minimized operational costs, and portability [4]. Microfluidic size-based separation devices can serve as valuable upstream tools for conditioning bead suspensions [5], removing aggregates [6], and isolating specific bead fractions [7] prior to downstream biological analysis [8], enabling high precision and reproducibility while reducing sample and reagent requirements.

Microfluidic sorting strategies can be briefly categorized into active and passive methods. Active approaches employ external fields (e.g., magnetic, electric [9], acoustic [10], or optical forces) to manipulate particles, but they often require complex setups and labeling. In contrast, passive methods exploit the intrinsic physical properties of particles, such as size, shape, and deformability, to achieve label-free separation. Among passive techniques, microflow approaches [11,12] have emerged as a particularly promising approach due to their ability to deliver high-throughput, size-based separation without external actuation. Furthermore, it has been recently reported that some novel passive microfluidic sorting strategies can be applied to different flow configurations, including single-phase inertial flows [13] and two-phase laminar flows [14].

Some recent particle separation techniques are provided in Table 1. Microflow focusing leverages hydrodynamic forces within microchannels to drive particles toward equilibrium positions determined by channel geometry and flow conditions [15]. Curved channels further enhance separation efficiency by introducing centrifugal effects and secondary flows (Dean vortices), thereby achieving differential focusing of particles based on size. Its core advantages lie in its extremely high throughput (often at the mL/min level), relatively simple structure, and capacity for label-free and continuous-flow operation [16]. However, this separation method has limited resolution, and its separation efficiency depends on flow rate and particle size. During operation, precise control is required, and a “flow rate sensitivity” issue arises [17]. Recent innovations have combined hydrodynamic focusing with deterministic lateral displacement (DLD) [18,19] using microstructures such as micropillars, enabling precise control of particle trajectories and improving resolution in size-based sorting. For instance, microspheres flowing near a cylindrical structure have been well studied [20]. Based on the lateral movements of particles from a specified upstream lateral position, the ideal location of micro-sieves can be predicted for sequential particle capture with a success rate of more than 90% [21]. The advantages include the precise size cutoff. Yet, the flow rate (typically at the μL/min level) is limited by the presence of microstructures, which are also prone to clogging. Sheath flow is sometimes required to pre-focus the sample [22]. Despite these advances, challenges remain in developing robust, scalable microfluidic systems for particle separation [23]. There is a demand for an integrated approach incorporating both inertial force and DLD, requiring more detailed optimization of channel architecture and flow dynamics [24] through theoretical modeling, computational simulation, and experimental validation [25,26].

In this work, we present a microfluidic device for size-based particle sorting, incorporating both inertial force and DLD strategies. It contains curved channels and straight channels embedded with cylindrical micropillars to achieve passive, continuous separation under laminar-flow conditions. Importantly, the device’s design minimizes the number of micropillars and thus hydraulic pressure, allowing a larger flow rate and greater throughput. Analysis of a spherical particle moving along a curved microchannel involves calculating equilibrium positions in different microchannel geometries and flow regimes. We performed theoretical analysis and numerical simulations to optimize channel design and micropillar arrangement, and then we conducted experimental validation using glass beads of different sizes. This study demonstrates the feasibility of combining the centrifugal phenomenon and DLD in a robust, user-friendly particle-sorting device, offering a foundation for future applications in biomedical diagnostics and cell isolation [27].

**Table 1:** Comparison of recently reported microfluidic particle separation methods.

Method	Pros	Cons	Size (μm)	Flow Rates (mL/min)	Separation Efficiency	Ref.
Inertial force	Simple structure and a high flow rate	Limited resolution, and dependence on flow rate and particle size	7.3, 9.9, 15.5	1–3	>90%	[16]
			12, 26	0.4	>85%	[28]
			10, 15, 20	0.18–0.27	>95%	[17]
DLD	Precise, and predictable size cutoff	Low flow rate and occurrence of clogging	4, 11	0.02–0.2	>98%	[22]
			2, 6	2 × 10^−4^	>90%	[29]
			10, 20	0.1–0.12	>89%	[30]
Inertial force and DLD	High flow rate, and precise size cutoff	More challenging design	5, 10, 20	N/A	>94%	[24]
			7, 13	0.05	>96%	[31]
			8, 15	0.2–0.6	>93%	This work

## 2. Materials and Methods

### 2.1. Device Fabrication

The device was fabricated based on soft photolithography. A silicon wafer was first spin-coated with a negative photoresist SU-8 (Kayaku Advanced Materials, Inc., Westborough, MA, USA), resulting in a uniform 40-micrometer-thick layer. Then, the coated wafer was heated on a hotplate at 95 °C for soft baking. After UV exposure and development, the patterned wafer served as a mold, with raised photoresist structures defining the microchannels. The device design is available in the Supplementary Materials. Polydimethylsiloxane (PDMS, SYLGARD™ 184, Midland, MI, USA) base and curing agent were mixed in a 10:1 weight ratio and then degassed in a vacuum jar. The degassed PDMS mixture was poured into a silicon wafer mold with a thickness of 3 mm. The mold was then placed in an oven and cured at 95 °C overnight to create the microchannel structures. Fluidic inlets and outlets on the PDMS substrate were created using a 1.6 mm diameter punch. Afterwards, the PDMS substrate was bonded onto a glass slide via air plasma.

### 2.2. Simulation

Three-dimensional (3D) models described in Cartesian coordinates were drawn with FreeCAD (ver. 1.0.1). These included (1) upstream and downstream curved channel sections and (2) straight channel sections into which micropillars were embedded with different lateral placement positions. 3D mesh generation of the model’s geometry was implemented using Gmsh, which is an open-source 3D finite-element mesh generator. The mesh/cell size was configured to have an average side length of ~2 μm. Numerical simulation of the flow profiles along the models were then performed using OpenFOAM (ver. 2.506). ‘Incompressible Flow Solvers’ was adopted with the default settings. The mass density and viscosity were set to constant values. No-slip conditions were set over the sidewalls (and micropillar surfaces), whereas a defined flowrate was set for the inlet/outlet of the channel sections. Afterward, the simulation results were exported using Paraview (ver. 5.11.2) for further analysis. The vector plot employs the nodal values, and velocity profiles over a selected cross-section were computed via bilinear interpolation with the nodal values obtained from the OpenFOAM simulation.

### 2.3. Image Capture

Microscopic images were captured using a phase-contrast inverted microscope (TE300, Nikon, Tokyo, Japan) with an sCMOS microscope camera (Andor Zyla 4.2, Andor, Oxford Instruments, Oxfordshire, UK).

### 2.4. Data Processing

Open-source image-processing software ImageJ ver. 1.54m (NIH) was used to analyze microscopic images.

### 2.5. Separation Efficiency

Glass beads (9000 Series Glass Particle Standards, Thermo Fisher Scientific, Waltham, MA, USA) of two distinct diameters (8 μm and 15 μm) were used to validate size-based separation. Bead suspensions were prepared at varying concentrations and introduced into the microfluidic device under controlled flow conditions. Separation efficiency was quantified by collecting output solutions from two outlets and counting beads using a hemocytometer. Separation efficiency (*E*) was calculated as follows:

(1) $$E=\frac{A_{1}+B_{2}}{A_{1}+A_{2}+B_{1}+B_{2}}$$

where *A*_1_ and *A*_2_ represent the number of 8 μm and 15 μm beads, respectively, in device outlet ‘1’, and *B*_1_ and *B*_2_ represent the number of 8 μm and 15 μm beads, respectively, in device outlet ‘2’.

### 2.6. Statistics

Error bars in plots represent standard errors. The *p*-values were determined via two groups of data using Student’s two-tailed, unpaired *t*-test. An asterisk in a plot represents a significant difference between two data groups (*p* < 0.05).

## 3. Results and Discussion

### 3.1. Device Design

**Figure 1:**
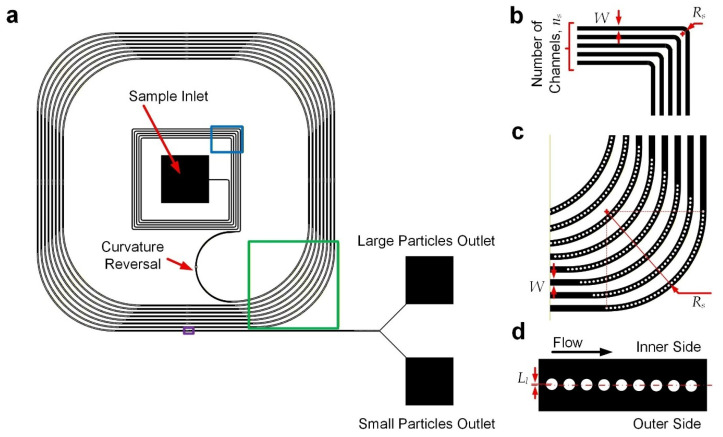
Device design. (**a**) Device layout. Part of the upstream turning channel region is highlighted with a *blue* box, part of the downstream curved channel is highlighted with a *green* box, and part of the downstream straight channel is highlighted with a *purple* box. (**b**) Structures of upstream turning channel region. (**c**) Structures of downstream curved channels. (**d**) Structures of downstream straight channels. Drawings may not be to scale.

The microfluidic particle separation device (Figure 1a) integrates two functional regions: an upstream curved channel section for initial lateral positioning of particles and a downstream curved channel section incorporating embedded cylindrical micropillars with a reversed direction of curvature for particle separation based on both inertial focusing and deterministic lateral displacement (DLD). To ensure there were consistent radii of channel curvature, the curved channels were composed of straight and quarter-circular arc channels, and hence the channel layout appears as squared paths with filleted corners. All the channel sections have a common height of 40 μm. The upstream channels (Figure 1b) have a width of 120 μm and a radius of curvature of 120 μm. It was expected that particles injected from the device inlet would gradually move to the outer cross-section along the upstream curved channels, pushed by centripetal force. A channel section with a reversed turning direction comes after the former section (Figure 1a). Next, there are downstream channels (Figure 1c) composed of repeating sets of straight channels with an opposite curvature direction (radius of curvature: 7200 μm) compared to the upstream ones, with a common width of 180 μm. Along these curved channels, particles should be located over the inner cross-section. Micropillars of 50 μm diameter were arranged at small enough intervals (center-to-center spacing: 75 μm), restricting lateral migration of the particles across the pillar array based on DLD. Furthermore, another series of nine micropillars were also placed along the straight channels (Figure 1d), with a center-to-center spacing of 75 μm along the channel and inward lateral displacements (*L_l_*) of 4, 3, 2, …, −4 μm such that the large particles would be laterally blocked by the pillars via DLD, while small particles located close to the pillars could traverse across to the outer half of the channels. Hence, the large and small particles will come out from the inner and outer outlets, respectively, as depicted in Figure 1a. This hybrid design combines inertial focusing and DLD principles to achieve continuous, size-based particle separation without external forces.

### 3.2. Inertial Lateral Displacement Along Curved Channels

**Figure 2:**
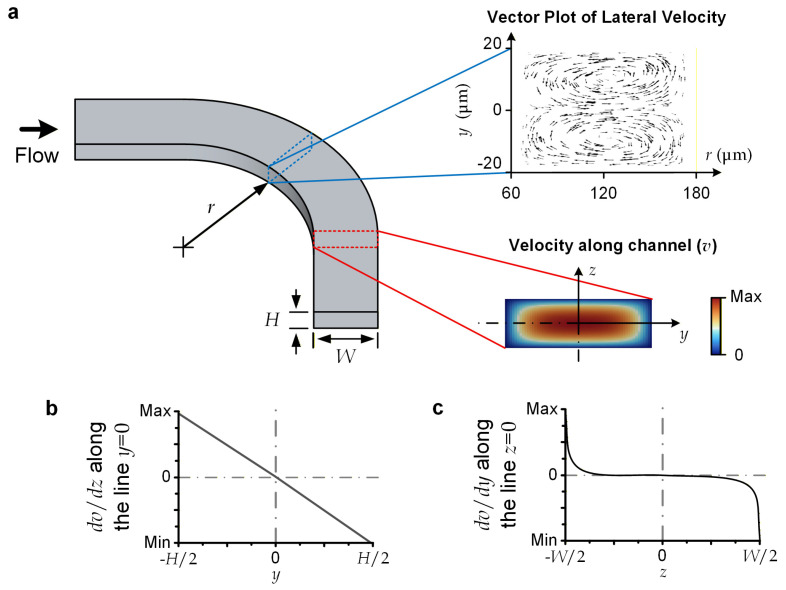
Flow characteristics. (**a**) Vector plot of lateral velocity profile, and axial velocity profile (*v*(*y*, *z*)) over channel cross-section. (**b**) *dv*/*dz* along the cross-sectional ‘vertical’ centerline, with *y* = 0. (**c**) *dv*/*dy* along the cross-sectional ‘horizontal’ centerline, with *z* = 0.

For the reported device, the microscale liquid flows should be laminar and have a sufficiently small Reynolds number (*Re*), which is defined as *Re = ρUH*/*μ* (<70), where *ρ* (=10^3^ kg/m^3^) is liquid density; *U* (<1.16 m/s) is the average flow velocity; *H* (=40 μm) is channel height, serving as the characteristic length in our case; and *μ* (=10^−3^ Pa·s) is liquid viscosity [32]. This flow regime induces highly predictable flow profiles [33]; and we have conducted numerical simulation to obtain the velocity profile for the upstream channel of the device (Figure 2a). Briefly, a major central region of the channel’s cross-section approaches a parabolic velocity profile along the channel height (*z*) direction. Considering that a lateral force is generated in the descending shear stress direction, a transiting particle should move laterally toward the cross-section line with *z* = 0 (Figure 2b) [34], where *y* = 0 and *z* = 0 constitute the center position of the cross-section. Notably, since the central range in the channel-width direction (*y*) has the same parabolic velocity as a function of *z*, the shear stress along *y* is negligible (Figure 2c). A transiting particle can still be stably displaced in the lateral direction under an external lateral force acting on it.

For the selected dimensions of microchannels, the centrifugal force is dominant over the Dean flow effects. For instance, the curved sections along the upstream channels (with a radius of curvature (*R_s_*) of 120 μm, *H* = 40 μm, and a channel width of 120 μm (or a hydraulic radius (*D_H_*) of 60 μm)) have the largest Dean flow effects over the entire flow region. This can be characterized by its Dean number (*De*), defined as *De* = *Re*·(*D_H_*/(2*R_s_*))^½^, with a value of 34.7, implying that the flow can be considered completely unidirectional (*De* < 40) [35]. We conducted a simulation to visualize the later velocity over the cross-section of the upstream curved channel (Figure 2a), indicating the maximum lateral velocity (~36.3 mm/s) is negligible compared with the axial velocity (*U* ~1 m/s). Therefore, we may simply consider the lateral displacement of a particle flowing along a curved channel (with a radius of curvature of *R_s_*) to be caused by the centrifugal effect alone. The centrifugal force (*F_c_*) becomes the drag force supporting the lateral movement of a spherical particle with a diameter of *a_p_* and a mass density of *ρ_p_*. *F_c_* can be expressed as follows:

(2) $$F_{c}=\frac{\pi {a_{p}}^{3}(\rho_{p}-\rho )U^{2}}{6R_{s}}$$

The lateral velocity (*V_c_*) of a particle should be proportional to the lateral force acting on the particle for low-Reynolds-number flow. *V_c_* can be expressed as follows:

(3) $$V_{c}\approx \frac{F_{c}}{3\pi \mu a_{p}}$$

Therefore, for a curved channel with a total turning length of *L_s_*, the lateral displacement (*D_L_*) of the particle at the exit of the channel can be approximated as follows:

(4) $$D_{L}\approx \frac{V_{c}L_{s}}{U}=\frac{(\rho_{p}-\rho ){{a_{p}}^{2}L}_{s}U}{18\mu R_{s}}$$

In principle, *L_s_*/*R_s_* ≈ 2*πn_s_*, where *n_s_* is the number of channel turns. *D_L_* is independent of *R_s_*, yet *D_L_* is proportional to *n_s_*. *D_L_* should be larger than half of the channel width (*W*) such that most particles displace to the outer side of the curved channel.

In this work, the upstream curved channels of the device are configured with dimensional parameters: *W* = 120 μm, *R_s_* = 120 μm, and *n_s_* = 5. In this work, we employed glass beads (*ρ_p_* = 2.5 kg/cm^3^) with a diameter (*a_p_*) of 15 μm (cat#9015, 9000 Series Glass Particle Standards, Thermo Scientific™) as the large particles and those with a diameter (*a_p_*) of 8 μm (cat#9008, Thermo Scientific™) as the small particles. (The microstructural dimensions and operation parameters can be reconfigured for other applications.) A flow rate of 0.0083 mL/s can induce *D_L_* > *W*/2 (114 μm for large particles and 72.7 μm for small particles), ensuring that most of the particles come out over the outer-half cross-section at the exit of the upstream curved channel region.

### 3.3. Deterministic Lateral Displacement Along Curved Channels in Which Micro-Pillars Are Embedded

Although large and small particles have different lateral velocities along curved channels, this difference is limited by a feasible channel length. Considering the hydraulic resistance along microchannels, we need to limit channel length such that the corresponding driving pressure lies below the binding strength of the channel substrates. To separate particles based on size, we add micropillar structures to introduce deterministic lateral displacement (DLD) along the downstream curved channels. Micropillars are placed at a defined distance away from each other, causing particles of different sizes to interact with them differently. Large particles can be kept on their original side because they cannot easily pass through the gaps, while small particles continue straight along with the flow of fluid. This separation happens naturally without needing external forces, making the method simple and passive.

The downstream channel has a width of 180 μm and a radius of channel curvature (*R_s_*) of 7.2 mm with eight turns (*n_s_*). It includes a series of micro-pillar obstacles (diameter (*R_o_*): 50 μm) with a center-to-center distance of 75 μm such that the effective channel width (*W_e_*) reduces to ~160 μm. By recalling Equation 2, we can then estimate that the total lateral displacement (*D_L_*) is 136 μm for large particles and 82.7 μm for small particles. The *D_L_* for both particle sizes is larger than *W_e_*/2, so most of the particles can drift by a pillar in any of the downstream curved channel sections. Based on DLD, the embedded micropillars can limit the lateral position to be barely next to these pillars, as opposed to passing across them. Hence, the closest lateral distance to the center of a micropillar can be approximated as ~*R_o_* + *a_p_*/2.

**Figure 3:**
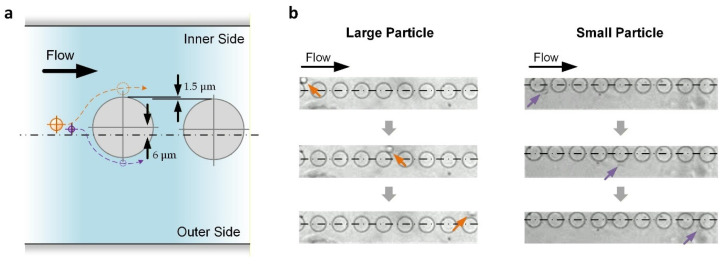
DLD of particles along the downstream straight channels in which micropillars are embedded. (**a**) Working principle. Only the first two pillars are shown. *Centerline* describes the central channel position. *Hidden lines* describe the expected trajectories of large (*orange*) and small (*purple*) particles. (**b**) Experimental results. *Centerlines* indicate the central channel position. Arrows indicate the large (*orange*) and small (*purple*) particles.

Transiting after the curved channel, the particles would then have a minimal lateral displacement of roughly equal to *a_p_*/2 × *W*/*W_e_*. For the reported device, this displacement is ~8.44 μm for large particles and ~4.5 μm for small particles. As depicted in Figure 3a, another series of nine micropillars (diameter: 50 μm; center-to-center distance: 75 μm) were placed along the following straight channels according to a method used in our previous analysis of micropillar placement for effective DLD [36]. The first pillar has an inward lateral displacement (*L_l_*) of 6 μm, which is between the minimal lateral displacements of the large and small particles, as mentioned above. Afterward, for each of the following pillars, *L_l_* reduces by 1.5 μm such that *L_l_* = −6 μm for the last pillar (outward). Based on DLD, all large particles are kept on the inner side of the flow channel, while small particles near the center of the channel can be pushed to the outer side of the flow channel (Figure 3b), achieving particle separation.

### 3.4. Experimental Validation

**Figure 4:**
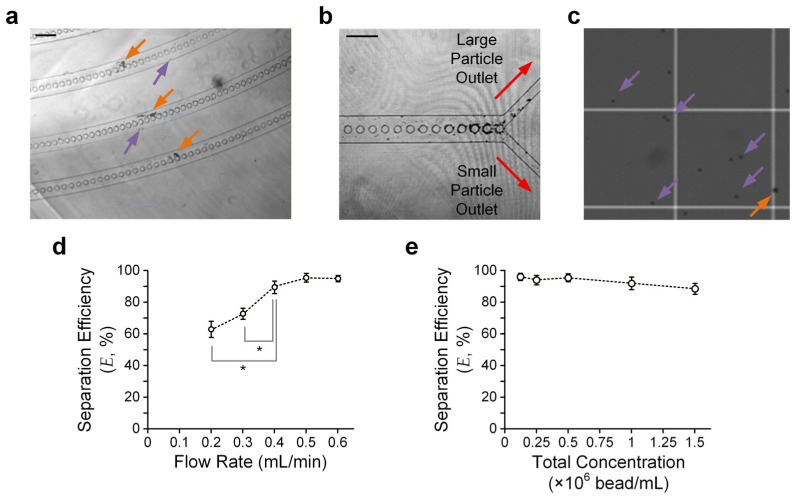
Particle separation results. (**a**) Sample micrograph taken in the downstream curved channels. *Orange* and *purple* arrows indicate large and small particles, respectively. Scale bar: 200 μm. (**b**) Sample micrograph taken near the device outlet channels. Scale bar: 200 μm. (**c**) Sample micrographs of sorted particles from ‘small particle outlet’ counted using a hemocytometer. (**d**) Separation efficiency (*E*) versus flow rate. Error bars are standard errors. (**e**) *E* versus total bead concentration. Three rounds of independent experimental repetitions were conducted. Error bars are standard errors. Asterisks indicate significant differences (*p* < 0.05) between the two comparison groups, highlighted by their adjacent lines (*gray*), determined using Student’s *t*-test.

To further verify the theoretical model, we conducted experiments in which beads were injected along the microfluidic particle separation device. We adopted glass beads with two different diameters (8 μm (small) and 15 μm (large)). We prepared a sample mixed with the two bead sizes with a defined bead density. In the experiments, we injected the sample via the device inlet at a defined flow rate driven by a syringe pump and collected the sorted beads from the device outlets. We expected the large beads to be present on the outer side of the downstream micro-pillar-embedded channels, and vice versa (Figure 4a). Hence, most large/small beads should have moved to the corresponding large/small particle outlet (Figure 4b). We then quantified separation efficiency (*E*), as defined in Equation 1, by counting the large and small particles using a hemocytometer under a microscope (Figure 4c).

We verified particle separation performance using a sample with the same density (2.5 × 10^5^ beads/mL) for both beads and quantified *E* for different flow rates, ranging from 0.2 mL/min to 0.6 mL/min, to obtain the optimal flow rate for separation. The results (Figure 4d) suggest that a flow rate of ≥0.5 mL/min can ensure *E* > 93%. The limitations for the further improvement of *E* may include the minor effects of Brownian motion, insufficient turns of the upstream/downstream turning channel region, and dimensional errors made during device fabrication.

We also conducted experiments to quantify *E* with a fixed flow rate (0.5 mL/min) but varying concentrations of beads. The bead concentration ranges from 0.125 to 1.5 × 10^6^ beads/mL. It can be observed that *E* is roughly maintained over such a concentration range (Figure 4e), agreeing with the working principle that the particle separation scheme is independent of the particle concentration. Yet, the particle concentration should not be so low that the hydrodynamic interactions between adjacent particles can be neglected. For instance, our results show that *E* > 93% only for a bead concentration of ≤5 × 10^5^ beads/mL.

Overall, these experiments demonstrate the promise of continuous, size-based particle separation using the reported device layout. Applying microchannel structures, combining inertial force and DLD, can lead to a separation efficiency of >93% without labeling. By configuring the device design for different ‘large’ and ‘small’ particles, the particle separation technique can potentially serve as a versatile tool for the separation of extracellular vesicles, microorganisms, mammalian cells, barcoded hydrogel particles, and engineered drug-delivery carriers [4,8]. For example, considering that many inertial microfluidic devices have been developed in recent years to purify bacteria for culture-free antimicrobial susceptibility [37], further optimizing the configuration presented in this work may allow separation of smaller particles with sizes comparable to those of bacteria. Moreover, particle separation is a critical process for isolating target populations from heterogeneous mixtures, which is essential for tasks such as circulating tumor cell (CTC) detection [38], hematopoietic stem cell purification, and fetal-cell isolation [39].

The cost-effective and passive sorting mechanism offers a promising alternative to more complex and expensive methods, making the technology accessible to a wider audience. Additionally, the modular design of the device allows for scalability and customization, ensuring its adaptability to various experimental needs.

## 4. Conclusions

This study presents a microfluidic device that achieves continuous, size-based particle separation by integrating inertial focusing and deterministic lateral displacement (DLD) within a compact channel architecture. The design combines upstream curved channels for initial lateral positioning with downstream micropillar-embedded curved channels to enable passive, label-free sorting under laminar-flow conditions. Theoretical analysis and numerical simulations confirmed the feasibility of the proposed mechanism, and experimental validation demonstrated a separation efficiency exceeding 93% for particles (glass) of two distinct sizes (8 μm and 15 μm) across a range of flow rates and concentrations. The device’s design can be reconfigured for other target particle groups.

The reported approach offers several advantages, namely, simplicity, cost-effectiveness, and scalability, making it suitable for diverse applications such as cell-based diagnostics, biomedical research, and environmental monitoring. Future work will focus on extending channel length and refining micropillar geometry to further enhance performance. By providing a tunable, label-free, and high-throughput means of separating microparticles, the presented device has the potential to support a wide range of biochemical, diagnostic, and cell-labeling applications.

## Data Availability

All data mentioned in the paper are provided here. More details can be provided on request.

## Supplementary Materials

The following supporting information can be downloaded at https://www.mdpi.com/article/10.3390/mi17020194/s1.
