# Supplementary figures and images for: Continuous Size-Based Particle Separation Using Inertial Force and Deterministic Lateral Displacement
Source: Micromachines (Basel). 2026 Jan 31;17(2):194. doi: 10.3390/mi17020194 (PMC12943751; doi:10.3390/mi17020194)

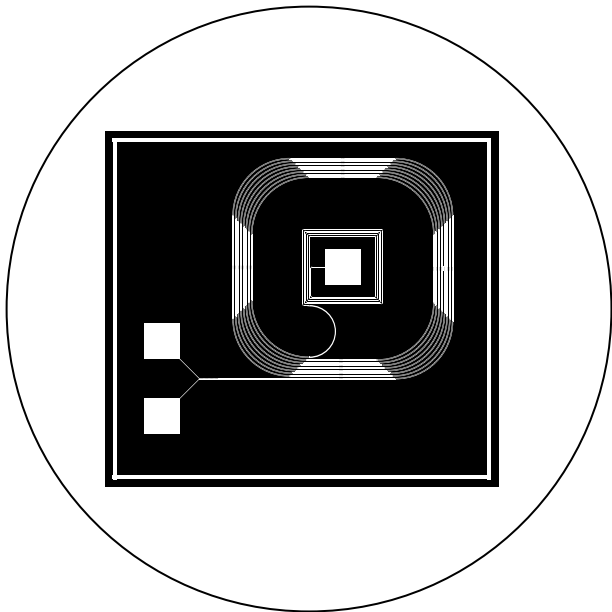

Supplement: Supplementary file 1 [file micromachines-17-00194-s001.zip › micromachines-4101770-supplementary.pdf]
